# 免疫检查点抑制剂治疗致非小细胞肺癌向小细胞肺癌组织学转化：病例报道及文献复习

**DOI:** 10.3779/j.issn.1009-3419.2025.101.12

**Published:** 2025-07-20

**Authors:** Xiting CHEN, Wenyuan HE, Ning YANG, Lijuan XIONG, Haoqiang WANG, Peng LIU, Bo XIE, Juan ZHOU

**Affiliations:** ^1^510006 广州，广州中医药大学研究生院; ^1^Guangzhou University of Chinese Medicine, Guangzhou 510006, China; ^2^510010 广州，中国人民解放军南部战区总医院病理科; ^2^Department of Pathology, General Hospital of Southern Theater Command, General Hospital of Southern Theater Command, Guangzhou 510010, China; ^3^510010 广州，中国人民解放军南部战区总医院肿瘤科; ^3^Department of Oncology, General Hospital of Southern Theater Command, Guangzhou 510010, China

**Keywords:** 免疫检查点抑制剂, 组织学转化, 肺肿瘤, Immune checkpoint inhibitors, Histological transformation, Lung neoplasms

## Abstract

非小细胞肺癌（non-small cell lung cancer, NSCLC）作为肺癌的主要组织学亚型，在所有肺癌病例中约占85%。近年来，以程序性死亡受体1/配体1（programmed death 1/programmed death ligand 1, PD-1/PD-L1）抑制剂为代表的免疫检查点抑制剂（immune checkpoint inhibitors, ICIs）在驱动基因阴性的NSCLC患者中取得了突破性进展，已被确立为一线治疗方案的重要组成部分并显著改善临床预后。然而，已有少量临床证据显示存在ICIs单药或联合治疗进展患者出现NSCLC向小细胞肺癌（small cell lung cancer, SCLC）组织学转化现象，而对于此类转化事件的临床特征、分子生物学基础及后续治疗策略目前仍缺乏系统性研究数据。本文报道了1例*KRAS*突变的肺腺癌患者经ICIs联合治疗16个月后发生SCLC转化的病例，并通过结合22例相似的文献病例进行了系统回顾。既往研究表明，小细胞转化是免疫治疗耐药的重要机制，转化后患者预后差。本文强调了治疗期间动态监测神经元特异性烯醇化酶（neuron-specific enolase, NSE）及规范二次活检的重要性，为临床实践提供了依据，有助于提高对这类少见的组织学转化的认知与应对能力，改善患者治疗效果。

## Abstract (en)

Non-small cell lung cancer (NSCLC), as the predominant histological subtype of lung cancer, accounts for approximately 85% of all lung cancer cases. In recent years, immune checkpoint inhibitors (ICIs), represented by programmed death 1/programmed death ligand 1 (PD-1/PD-L1) inhibitors, have achieved breakthrough advancements in patients with driver gene-negative NSCLC. They have been established as a key component of first-line treatment regimens and have significantly improved clinical outcomes. However, limited clinical evidence has emerged showing the phenomenon of histological transformation from NSCLC to small cell lung cancer (SCLC) in patients experiencing disease progression after ICIs monotherapy or combination therapy. Systematic research data on the clinical characteristics, molecular biological basis, and subsequent treatment strategies for such transformation events are currently lacking. This article reports a case of SCLC transformation occurring in a patient with *KRAS*-mutated lung adenocarcinoma after 16 months of ICIs combination therapy and provides a systematic review of 22 similar published cases. The study demonstrates that small cell transformation is a critical mechanism of immunotherapy resistance, and transformed patients exhibit poor prognosis. The research emphasizes the importance of dynamic monitoring of neuron-specific enolase (NSE) and standardized repeat biopsies during treatment, providing a basis for clinical practice. This aids in enhancing the recognition and management capabilities for this rare histological transformation, ultimately improving patient outcomes.

在全球范围内，肺癌已成为癌症发病率及死亡率均居前列的恶性肿瘤^[1]^。肺癌根据病理特征主要分为非小细胞肺癌（non-small cell lung cancer, NSCLC）和小细胞肺癌（small cell lung cancer, SCLC）^[2]^，两种肺癌在起源、发病率、生物学行为、分子特点及治疗预后等各个方面均存在显著不同。得益于以分子靶向药物及免疫检查点抑制剂（immune checkpoint inhibitors, ICIs）为代表的新型药物在NSCLC中的应用，NSCLC的预后明显提高，5年总体生存（overall survival, OS）率可达26.4%^[3]^，而SCLC患者的药物治疗疗效及预后仍有待提高。

表皮生长因子受体（epidermal growth factor receptor, EGFR）突变是NSCLC最常见的驱动基因之一，针对EGFR的酪氨酸激酶抑制剂（tyrosine kinase inhibitors, TKIs）已经成为此类患者的一二线标准治疗，但在用药10-21个月后均会发生获得性耐药，经再次活检证实SCLC转化是EGFR-TKIs获得性耐药机制之一，发生率为3%-14%^[4,5]^。近年来，随着ICIs在驱动基因*EGFR*/间变性淋巴瘤激酶（anaplastic lymphoma kinase, *ALK*）阴性NSCLC中的广泛应用，对ICIs单药或者联合治疗进展的患者也有一部分表现出SCLC转化，提示组织转化也可能是ICIs重要耐药机制之一，但对于具体的临床病例特征、分子生物学改变、后续治疗选择等目前仍缺少数据。因此，本文通过对1例*KRAS* 突变的肺腺癌患者，经ICIs联合治疗约16个月后发生SCLC转化的病例进行报道及相关文献回顾^[2,6-20]^，系统总结了NSCLC患者免疫治疗后发生SCLC转化的特点，为此类患者临床实践与科学研究提供依据。

## 1 病例资料

患者男，70岁，吸烟40余年，1包/d。于2023年10月因“食欲减退伴消瘦半年余”就诊，经影像学及病理学检查确诊为肺腺癌（IV期），伴脑、骨、肝、肾等多发转移，确诊后患者戒烟。免疫组化（图1）提示甲状腺转录因子1（thyroid transcription factor-1, TTF-1）（+），CD56（-），CgA（-），突触核蛋白（Synuclein, Syn）（-），Napsin A（部分+），CK5/6（-），CK7（+），P40（个别+），P53（-），P63（局灶+），EGFR（+），ALK（-），细胞增生核抗原（ki-67 antigen, Ki67）约为50%，程序性死亡配体1（programmed death ligand 1, PD-L1）肿瘤细胞阳性比例分数（tumor proportion score, TPS）=90%。基因检测显示：微卫星稳定（microsatellite-stable, MSS），KRAS p.G12C突变，CDKN2缺失，肿瘤突变负荷（tumor mutational burden, TMB）高达27.56 Muts/Mb。

**图1:**
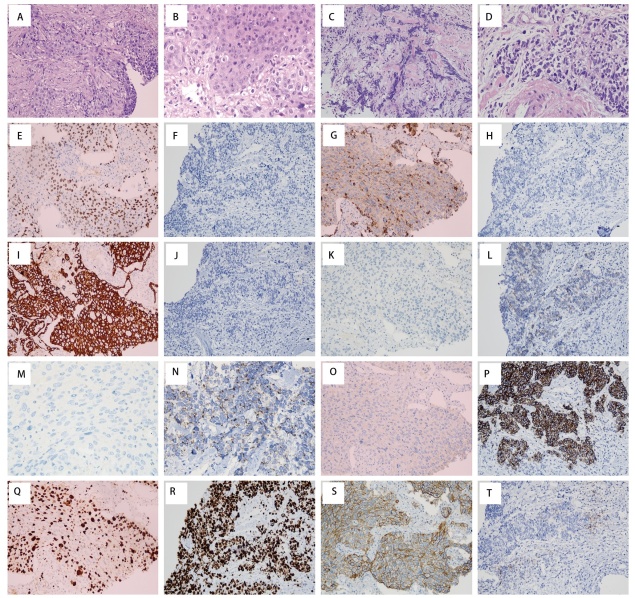
病理图片。A：肺腺癌肿瘤细胞体积较大，呈巢片状浸润性生长（HE，×200）；B：腺癌肿瘤细胞胞界不清，细胞核大、核仁明显，胞质丰富嗜酸，核分裂象易见（HE，×400）；C：小细胞肺癌细胞体积较小，呈条索状或散在浸润性生长（HE，×200）；D：小细胞肺癌细胞黏附性差，细胞核染色质呈纤细颗粒状，无明显核仁，胞质稀少，核分裂象易见（HE，×400）；E、F：TTF-1肺腺癌细胞核阳性，小细胞肺癌阴性（免疫组化EnVision法，×200）；G、H：Napsin A肺腺癌细胞质阳性，小细胞肺癌阴性（免疫组化EnVision法，×200）；I、J：CK7肺腺癌细胞质阳性，小细胞肺癌阴性（免疫组化EnVision法，×200）；K、L：CD56肺腺癌阴性，小细胞肺癌细胞膜阳性（免疫组化EnVision法，×200）；M、N：CgA肺腺癌阴性，小细胞肺癌细胞核旁点状阳性（免疫组化EnVision法，×400）；O、P：Syn肺腺癌阴性，小细胞肺癌细胞质阳性（免疫组化EnVision法，×200）；Q、R：Ki67肺腺癌肿瘤细胞约50%细胞核阳性，小细胞肺癌肿瘤细胞约95%细胞核阳性（免疫组化EnVision法，×200）；S、T：PD-L1肺腺癌90%肿瘤细胞细胞膜阳性，小细胞肺癌2%肿瘤细胞细胞膜阳性（免疫组化EnVision法，×200）。

2023年10月18日，患者行枕部脑转移病灶伽马刀治疗，2023年10月20日至2024年2月26日，行7个周期“培美曲塞0.6 g+贝伐珠单抗300 mg+信迪利单抗200 mg，每3周1个疗程”联合治疗。治疗期间每2个周期依据实体瘤疗效评价标准1.1版（Response Evaluation Criteria in Solid Tumors version 1.1, RECIST 1.1）评估疗效，最佳疗效为部分缓解（partial response, PR）（图2）。2024年2月28日，因肌酐进行性升高（最高278 μmol/L），改为信迪利单抗免疫维持治疗，后肌酐水平部分恢复（145-157 μmol/L）。信迪利单抗免疫维持治疗至2025年1月15日，最佳疗效评估为疾病稳定（stable disease, SD）。2025年2月8日因肿瘤标志物神经元特异性烯醇化酶（neuron-specific enolase, NSE）显著升高（180.10 μg/L）（图3），进一步行影像学检查提示腹腔、腹膜后转移病灶进展增多增大，评价肿瘤进展（progressive disease, PD）（图2），再次活检病理证实为转移性小细胞癌，免疫组化（图1）提示：TTF-1（-），CD56（+），CgA（+），Syn（+），Napsin A（部分-），CK5/6（-），P63（-），Ki67（95%+），PD-L1 TPS=2%，再次基因检测：MSS，KRAS p.G12R突变，CCNE1扩增，出现TP53和RB1突变。考虑到患者肾功能不全，给予依托泊苷化疗1个疗程，化疗后患者症状未缓解。2025年3月11日出现肌红蛋白高、肾功能不全加重、高尿酸血症、肺部感染等不适，经治疗效果欠佳。2025年3月16日患者死亡。自确诊肺癌后OS约为17个月，自确诊小细胞转化OS仅1.5个月。

**图2:**
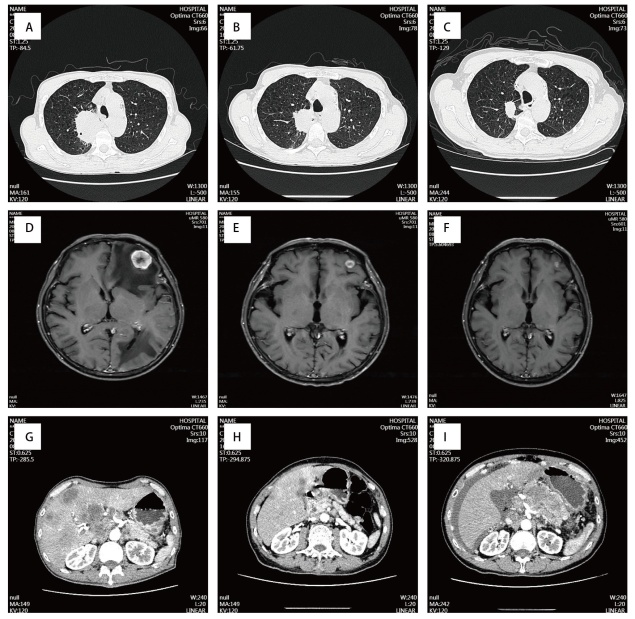
患者治疗及小细胞转化前后的影像学图片。A：2023-10-09初始治疗时，右肺上叶后段可见一不规则团块状软组织密度灶；B：2023-11-29免疫治疗2个周期后，肺部病灶缩小；C：2025-02-12小细胞转化后，肺部病灶仍稳定缩小；D：2023-10-13初始治疗时，左侧额叶可见环状不规则强化病灶，周围可见片状水肿；E：2023-11-30免疫治疗2个周期后，脑转移灶缩小；F：2025-02-08小细胞转化后，脑部病灶仍稳定缩小；G：2023-10-09初始治疗时，肝实质内可见多个团块状混杂强化灶；H：2023-11-29免疫治疗2个周期后，肝脏病灶消退，胰腺周围未见病灶；I：2025-02-12小细胞转化后，肝脏病灶未见进展，腹膜后胰腺周围多发淋巴结转移，合并腹水。

**图3:**
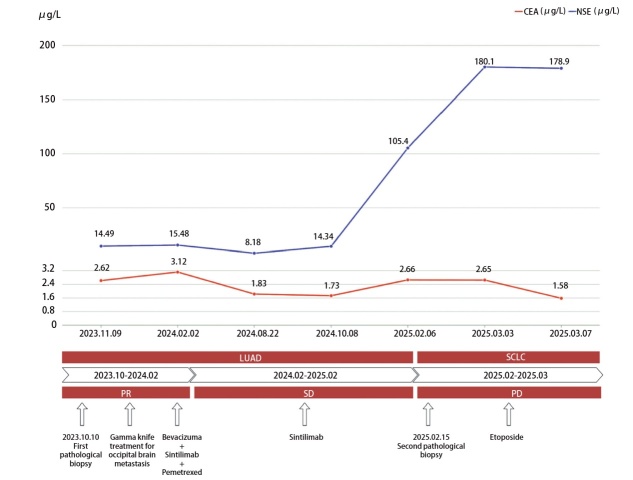
CEA、NSE变化趋势

## 2 讨论

本例患者初诊为晚期肺腺癌（IV期，*KRAS*突变，PD-L1高表达，TMB-H），符合免疫治疗优势人群的特征。初始接受免疫联合化疗方案，最佳疗效为PR，在ICIs治疗16个月后，患者原发病灶保持稳定，但腹腔转移灶迅速进展，经腹腔病灶活检病理证实发生小细胞转化。除本文报道的1例外，我们对2017年至2024年发表的16篇文献中报告的22例NSCLC在免疫治疗后发生SCLC转化的病例进行了总结（表1），其中，国外病例14例，国内8例，患者以中老年男性为主，中位年龄65岁，男性占比68%，普遍具有长期吸烟史。初诊时大部分为不可手术切除的局部晚期或晚期转移患者，病理类型以肺腺癌和肺鳞癌为主，大部分为驱动基因*EGFR*/*ALK*阴性患者，有2例EGFR突变患者在使用EGFR-TKIs后进展，二线使用化疗联合ICIs后出现SCLC转化。驱动基因*EGFR*/*ALK*阴性的晚期NSCLC患者一线方案为化疗联合ICIs，大部分为PD-1抗体，国外病例主要使用纳武利尤单抗和帕博利珠单抗，国内病例主要使用纳武利尤单抗、帕博利珠单抗以及国产的信迪利单抗、特瑞普利单抗，有1例患者使用了针对PD-L1的舒格利单抗。患者在使用ICIs后2-34个月（中位15.5个月）出现病情进展，并通过再次活检证实SCLC转化，但目前由于数据量少，还未能显示哪种ICIs更容易诱导组织学转化。

**表1:** 23例免疫检查点抑制剂诱导转化的小细胞肺癌临床特征及治疗策略

Author	Age (yr)	Sex	Smoking history	Initial pathological type	Whether to perform second genetic test	NSE after transformation	Treatment before transformation	Treatment after transformation	Duration of immune therapy before transformation	PFS after transformation	OS after transformation
Imakita T, et al.^[6]^	75	M	50 pack-years	NSCLC	Unknown	Elevated	Chemotherapy+ Immunotherapy	Chemotherapy	4 months	Unknown	2 months
Abdallah N, et al.^[2]^	65	M	35 pack-years	LUAD	Unknown	Unknown	Chemotherapy+ Immunotherapy	Chemotherapy	4 months	Unknown	Unknown
	68	M	Unknown	LUSC	Unknown	Unknown	Chemotherapy+ Immunotherapy	Chemotherapy+ Radiotherapy	25 months	Unknown	Unknown
Bar J, et al.^[7]^	70	F	Current	LUSC	Yes， TP53 mutation	Unknown	Chemotherapy+ Immunotherapy	Immunotherapy	16 months	8 months	Unknown
	75	M	Past (>10 years)	LUSC	Yes， TP53 mutation	Unknown	Chemotherapy+ Immunotherapy	Chemotherapy+ Immunotherapy +Targeted therapy+ Radiotherapy	7 months	1 month	13 months
Iams WT, et al.^[8]^	67	F	50 pack-years	LUAD	Yes， TP53 and RB1 mutations	Unknown	Chemotherapy+ Immunotherapy	Chemotherapy	16 months	2 months	11 months
	75	F	30 pack-years	LUAD	Yes， TP53 mutation	Unknown	Chemotherapy+ Immunotherapy	Chemotherapy+ Immunotherapy	24 months	4 months	16 months
Okeya K, et al.^[9]^	66	M	45 pack-years	LUAD	Unknown	Elevated	Chemotherapy+ Immunotherapy	Chemotherapy	6 months	3 months	5 months
Miura N, et al.^[10]^	65	M	34 pack-years	LUAD	Unknown	Elevated	Immunotherapy	Chemotherapy+ Radiotherapy	4 months	Unknown	17 months
Sehgal K, et al.^[11]^	mid- 60s	F	35 pack-years	LUSC	Yes， TP53 mutation	Unknown	Chemotherapy+ Immunotherapy	Chemotherapy+ Immunotherapy +Radiotherapy	21 months	11 months	14 months
Si X, et al.^[12]^	69	M	48 years	LUSC	Yes， TP53 mutation	Elevated	Immunotherapy	Chemotherapy	15 months	Unknown	Unknown
Imakita T, et al.^[13]^	64	M	84 pack-years	LUSC	Yes	Decreased	Chemotherapy+ Immunotherapy	Unknown	34 months	Unknown	Unknown
	74	F	Never	LCNEC	Unknown	Decreased	Chemotherapy+ Immunotherapy	Unknown	18 months	Unknown	Unknown
	70	M	88 pack-years	LUSC	Unknown	Unknown	Chemotherapy+ Immunotherapy	Unknown	7 months	Unknown	Unknown
Shen Q, et al.^[14]^	69	M	30 pack-years	LUSC	Unknown	Elevated	Immunotherapy	Best supportive care	2 months	Unknown	2 months
	71	M	34 pack-years	LUSC	Unknown	Elevated	Chemotherapy+ Immunotherapy	Chemotherapy	2 months	2 months	6 months
Zhai X, et al.^[15]^	43	M	Never	LUAD	Yes， TP53 and RB1 mutations	Unknown	Chemotherapy+ Immunotherapy	Chemotherapy+ Immunotherapy +Targeted therapy	21 months	Unknown	21 months
Liu H, et al.^[16]^	75	M	40 pack-years	LUSC	Yes	Unknown	Chemotherapy+ Immunotherapy	Immunotherapy	17 months	Unknown	Unknown
Yang M-H, et al.^[17]^	50	M	20 years	LUAD	Yes	Unknown	Chemotherapy+ Immunotherapy	Chemotherapy+ Targeted therapy	7 months	2 months	Unknown
Wang D, et al.^[18]^	56	M	30 years	LUSC	Unknown	Elevated	Chemotherapy+ Immunotherapy	Chemotherapy	7 months	0 month	6 months
Li Q, et al.^[19]^	67	F	20 years	LUSC	Unknown	Unknown	Immunotherapy	Chemotherapy	3 months	Unknown	Unknown
Tomic K, et al.^[20]^	65	F	20 pack-years	LUAD	Unknown	Unknown	Immunotherapy	Chemotherapy+ Radiotherapy	25 months	7 months	11.5 months
Case in this research	70	M	Unknown	LUAD	Yes， TP53 and RB1 mutations	Elevated	Chemotherapy+ Immunotherapy	Chemotherapy	15.5 months	0 month	1.5 months

M: male; F: female; NSCLC: non-small cell lung cancer; LUSC: lung squamous cell carcinoma; LCNEC: large-cell neuroendocrine carcinoma; PFS: progression-free survival; OS: overall survival.

SCLC病理转化后呈现典型的神经内分泌分化特征，表现为CD56、Syn、CgA等标志物普遍阳性。既往研究^[21,22]^表明，ICIs治疗期间，NSCLC疾病超进展（hyperprogressive disease, HPD）发生率为8%-14%。尽管其机制尚未完全阐明，但本例患者在接受ICIs治疗期间出现疾病快速进展，经穿刺活检证实发生SCLC转化，该病理学转化特征与Okeya等^[9]^报道的病例相似，提示SCLC转化可能是ICIs治疗诱发HPD的潜在机制之一。因此，对于ICIs治疗耐药后出现HPD的患者，应考虑组织学转化的可能性。同样值得注意的是，转化后患者NSE水平普遍显著升高，提示其可作为监测转化的血清学标志，有研究^[23]^报道NSCLC患者治疗过程中出现NSE的显著升高提示SCLC转化可能。因此，对NSE血清水平的常规和动态检测可能有助于在侵入性活检之前筛选出SCLC转化的患者。

当前关于NSCLC向SCLC转化的机制尚未完全明确。一方面，有研究^[4]^推测，NSCLC和SCLC可能源自具有多向分化潜能的呼吸道上皮祖细胞（尤其是II型肺泡上皮细胞）。在特定致癌驱动因素（如EGFR突变）和肿瘤微环境压力的影响下，可能通过表观遗传重编程或转分化机制，实现从腺癌细胞表型向神经内分泌特征的SCLC表型的转变。另一方面，也有学者^[24]^提出，部分患者初诊时肿瘤本身即同时存在SCLC与NSCLC两种成分，在初次活检时，可能仅检测到NSCLC成分，但在手术切除标本或尸检中却发现同时存在SCLC与NSCLC的混合组织学特征。在治疗选择的压力下，对治疗耐药的SCLC逐渐作为优势组分发生克隆扩增，主导后续肿瘤进展。此外，NSCLC向SCLC的转化过程可能涉及一类具有自我更新能力和多向分化潜能的肿瘤干细胞亚群^[25]^，在治疗压力的持续选择下，这类干细胞本身具备了分化为神经内分泌肿瘤细胞的固有潜能。药物压力不仅筛选出具有耐药性的干细胞克隆，还通过诱导染色质可塑性及转录重编程，促使干细胞加速获得神经内分泌标志物表达。当前与组织学转化相关的临床报道正日益增多，然而关于转化后的肿瘤应被视为新成分还是其本就是复合型肿瘤这一问题仍存在争议。Wang等^[26]^利用全外显子测序，分析了*TP53*、*RB1*、*EGFR*在复合型神经内分泌癌中的分子改变和克隆演化，发现不同成分的肺癌细胞具有共同的克隆起源，这一发现提示了对转化前后*TP53*、*RB1*、*EGFR*等基因状态进行评估，将有助于将潜在的组织转化病例与原本就是复合型肺癌病例区分开来。此外，药物压力下抑癌基因RB1和TP53的缺失突变^[27]^也是构成患者SCLC转化的分子基础和必要条件。目前，上述机制探讨主要基于EGFR-TKIs耐药致SCLC转化的研究，而ICIs致SCLC转化的具体机制方面仍有待进一步探索。在本例及我们所回顾的22例ICIs致SCLC转化患者中，8例有再次基因检测的患者均出现了TP53和/或RB1的突变，证实此类基因的改变也是ICIs致SCLC转化的机制之一。

与接受EGFR-TKIs治疗后常规开展二次活检不同，晚期NSCLC患者在接受ICIs治疗期间鲜少进行重复组织采样，这导致ICIs治疗后发生小细胞转化的实际发生率可能被严重低估^[11]^。这一现状或致使此类患者的诊疗面临困境：临床医生若缺乏对小细胞转化风险的认知，可能延误二次病理活检，从而使患者群体无法及时接受针对小细胞转化的精准治疗。目前，SCLC转化后的治疗方案多遵循SCLC治疗指南，首选铂类联合依托泊苷方案化疗（联合或不联合ICIs），其他方案包括蒽环类、紫杉类化疗药物。然而尽管上述治疗方案可短期内控制病情进展，但疗效持续时间有限：转化后中位无进展生存期（progression-free survival, PFS）多维持在2.4-5.4个月^[5,28,29]^，转化后的OS约为10个月^[29,30]^，这一生存数据与之前报道的*EGFR*突变型肺腺癌在EGFR-TKIs耐药后发生SCLC转化者的OS为10.9个月（95%CI：8.0-13.7个月）相当，与广泛期SCLC总体生存预后相比也基本类似^[29]^。

综上，本文通过1例病例报道及文献复习探索了ICIs治疗NSCLC过程中发生SCLC转化的临床特征及机制。现有证据表明，SCLC转化不仅是免疫治疗耐药的重要机制，更是肿瘤通过表型转化实现免疫“逃逸”的关键进化策略。这种适应性进化策略使得肿瘤细胞既能规避免疫监视，又可利用SCLC固有的高增殖特性实现快速进展。临床实践中，由于ICIs治疗后SCLC转化发生率被严重低估，ICIs治疗期间的转化预警体系亟待建立：一方面需强化利用血清NSE动态监测等作为无创筛查工具的意识，另一方面应规范进展期患者二次活检的检查流程，从而为病理转化患者争取更精准更及时的治疗时机。而对于SCLC转化机制的阐明对优化治疗策略同样具有重要的指导价值，可为预防或延缓转化的联合治疗方案探索效果更佳的路径。

本病例已获得患者家属知情同意，相关内容通过所在机构伦理委员会审批（伦理号：NZLLKZ2024159）。
